# In Vitro Insights into the Dietary Role of Glucoraphanin and Its Metabolite Sulforaphane in Celiac Disease

**DOI:** 10.3390/nu16162743

**Published:** 2024-08-17

**Authors:** Elisa Sonzogni, Giulia Martinelli, Marco Fumagalli, Nicole Maranta, Carola Pozzoli, Corinne Bani, Luigi Alberto Marrari, Chiara Di Lorenzo, Enrico Sangiovanni, Mario Dell’Agli, Stefano Piazza

**Affiliations:** 1 Department of Pharmacological and Biomolecular Sciences “Rodolfo Paoletti” (DiSFeB), Università Degli Studi di Milano, 20133 Milan, Italy; elisa.sonzogni@unimi.it (E.S.); giulia.martinelli@unimi.it (G.M.); marco.fumagalli3@unimi.it (M.F.); nicole.maranta@unimi.it (N.M.); carola.pozzoli@unimi.it (C.P.); corinne.bani@unimi.it (C.B.); chiara.dilorenzo@unimi.it (C.D.L.); mario.dellagli@unimi.it (M.D.); stefano.piazza@unimi.it (S.P.); 2 Naturalsalus S.r.l., Trezzano Rosa, 20060 Milan, Italy

**Keywords:** glucosinolates, glucoraphanin, sulforaphane, gut inflammation, gut barrier, celiac disease

## Abstract

Sulforaphane is considered the bioactive metabolite of glucoraphanin after dietary consumption of broccoli sprouts. Although both molecules pass through the gut lumen to the large intestine in stable form, their biological impact on the first intestinal tract is poorly described. In celiac patients, the function of the small intestine is affected by celiac disease (CD), whose severe outcomes are controlled by gluten-free dietary protocols. Nevertheless, pathological signs of inflammation and oxidative stress may persist. The aim of this study was to compare the biological activity of sulforaphane with its precursor glucoraphanin in a cellular model of gliadin-induced inflammation. Human intestinal epithelial cells (CaCo-2) were stimulated with a pro-inflammatory combination of cytokines (IFN-γ, IL-1β) and in-vitro-digested gliadin, while oxidative stress was induced by H_2_O_2_. LC-MS/MS analysis confirmed that sulforaphane from broccoli sprouts was stable after simulated gastrointestinal digestion. It inhibited the release of all chemokines selected as inflammatory read-outs, with a more potent effect against MCP-1 (IC_50_ = 7.81 µM). On the contrary, glucoraphanin (50 µM) was inactive. The molecules were unable to counteract the oxidative damage to DNA (γ-H2AX) and catalase levels; however, the activity of NF-κB and Nrf-2 was modulated by both molecules. The impact on epithelial permeability (TEER) was also evaluated in a Transwell^®^ model. In the context of a pro-inflammatory combination including gliadin, TEER values were recovered by neither sulforaphane nor glucoraphanin. Conversely, in the context of co-culture with activated macrophages (THP-1), sulforaphane inhibited the release of MCP-1 (IC_50_ = 20.60 µM) and IL-1β (IC_50_ = 1.50 µM) only, but both molecules restored epithelial integrity at 50 µM. Our work suggests that glucoraphanin should not merely be considered as just an inert precursor at the small intestine level, thus suggesting a potential interest in the framework of CD. Its biological activity might imply, at least in part, molecular mechanisms different from sulforaphane.

## 1. Introduction

Glucosinolates are glycosidic metabolites containing sulfur and nitrogen, occurring in plants belonging to the Brassicaceae family. Edible cultivars from *Brassica oleracea* species are consumed all over the world in the form of cabbage, broccoli, and cauliflower, thus representing a relevant source of glucosinolates for humans [1]. The main vegetable sources cited by the literature are broccoli sprouts and seeds (*Brassica oleracea* var. italica Planck), although these compounds are also present in mature tissues of the plant in lower amounts [2].

The main glucosinolate found in broccoli is glucoraphanin, which is biotransformed into the isothiocyanate sulforaphane by the action of myrosinase, an enzyme occurring in plants and bacteria, including the microbiota [2]. It is considered a biologically active compound with relevant interest for human health. Several authors have collected the evidence concerning its potential role in cancer and cardiometabolic prevention [3,4,5]. The protective effect of *Brassica oleracea* cultivars on colon cancer has been suggested by epidemiological and pre-clinical studies [6,7,8,9]. According to many authors, sulforaphane represents the main compound responsible for the effect against colon cancer, due to its well-established anti-proliferative, antioxidant, and anti-inflammatory properties [3,8,9].

Nevertheless, the impact of glucosinolates on intestinal disorders, with specific reference to inflammatory-based diseases, is still unclear. Several authors have already revised the recent literature concerning the effect of broccoli sprouts in rodent models of colitis [10,11], thus remarking on the crucial role of glucosinolate content. The same authors suggested that more studies on intestinal inflammation with different origins are needed to encourage the translation to clinical trials [11].

Of note, glucoraphanin is frequently mentioned as a stable and inert pro-active compound, which requires its bioconversion into sulforaphane to exert its effects at both the intestinal and systemic levels [2]. Accordingly, the literature concerning isothiocyanates focuses on sulforaphane as a prototypic and bioactive structure, while biological comparison with the precursor glucoraphanin is rare [5]. On the other hand, it is important to consider that glucoraphanin could remain stable as it passes through the gastrointestinal tract reaching the large intestine [12], thus raising the hypothesis regarding its biological activity at both the gastric and small intestine levels.

Considering the metabolic fate of glucosinolates, we speculated on the potential role of glucoraphanin in inflammatory conditions affecting the small intestine, such as celiac disease (CD). In line with other intestinal disorders, active CD was correlated with impaired intestinal barrier and dysbiosis [13,14]. Gliadin plays a role as either an immunogenic hapten or a pro-inflammatory agent in CD: the inflammatory effectors involved in the autoimmune process have been described, with innate cytokines (such as IL-1β) and IFN-γ playing a well-established role [15]; on the contrary, the inflammatory pathways involved in the direct effect of gliadin on enterocytes are still unclear. According to the literature, they might include the NF-κB pathway [16], the JAK/STAT pathway [17], and the interaction with receptors for CXC- chemokines, such as CXCR3 [18].

Independently of the autoimmune background of CD, several studies have also suggested that intestinal homeostasis might be directly altered by undigested peptides from gliadin, which can induce oxidative stress in different experimental models: for example, gliadin was involved in DNA damage at the intestinal level, since γ-H2AX resulted in phosphorylation in both in vitro and histological studies [19].

Despite the severe outcomes of CD, which are successfully prevented by the exclusion of gluten from the diet, oxidative and inflammatory markers might persist in patients [15,20,21]. Our previous works sustained the potential value of antioxidant compounds from gluten-free foods, such as pigmented cereals, for healthy diets dedicated to celiac people [22,23]. Accordingly, the present work aims to compare the biological effect of glucoraphanin and sulforaphane in a model of human intestinal epithelium (CaCo-2 cells), challenged by gliadin and pro-inflammatory cytokines involved in autoimmune diseases. Ultimately, this study wonders whether cruciferous vegetables might be rationally included in gluten-free diets due to their glucosinolate content.

## 2. Materials and Methods

### 2.1. Materials

Methanol, ethanol, and HPLC-grade water were from VWR International (Fontenay-sous-Bois, France). Gliadin, digestive enzymes, sodium butyrate, and sulforaphane were purchased from Sigma-Aldrich (Merck Life Science, Milano, Italy). Glucoraphanin, apigenin, and resveratrol were purchased from PhytoLab GmbH & Co. KG (Vestenbergsgreuth, Germany). Hydrogen peroxide 30% (*w*/*w*) (H_2_O_2_), containing stabilizer, was purchased from Sigma-Aldrich (Merck Life Science, Milano, Italy) and stored at +4 °C, according to manufacturer instructions. Natural compounds were dissolved by DMSO at the concentration of 20 mM and stored at −20 °C until cell treatments.

Materials for cell culture included high-glucose DMEM from Sigma-Aldrich (Merck Life Science, Milano, Italy); RMPI medium, Trypsin-EDTA 0.25%, streptomycin, penicillin, non-essential amino acids, sodium pyruvate, L-glutamine from Gibco^TM^ (Thermo Fisher Scientific, Monza, Italy); fetal bovine serum (FBS) and disposable materials (Primo^®^ or Falcon^®^) were from Euroclone (Euroclone S.p.a., Pero, Italy) or Corning Life Sciences (Amsterdam, The Netherlands). Bicinchoninic acid (BCA) assay was also from Euroclone.

Cytokines and ELISA kits were purchased from Peprotech (PeproTech Inc., London, UK); plasmid transfection kit and CM-H2DCFDA were from Invitrogen^®^ (Thermo Fisher Scientific, Monza, Italy), while Britelite^TM^ Plus reagent was from Perkin Elmer (Perkin Elmer Milano, Italy).

### 2.2. Cell Culture

Human intestinal epithelial cells from colorectal adenocarcinoma (CaCo-2, clone HB237; ATCC, Manassas, VA, USA) were cultured in high-glucose DMEM containing 100 mg streptomycin, 100 units penicillin, 1% non-essential amino acids, 1 mM sodium pyruvate, 4 mM L-glutamine, and 10% FBS. During the adherent growth in 75 cm^2^ flasks, cells were incubated under a humidified atmosphere with 5% CO_2_ at 37 °C. After 48 h or 72 h, avoiding confluency, cells were detached by Trypsin-EDTA 0.25%, counted, and placed into new flasks, or seeded in adequate experimental supports for another 48 h.

When specified, cells were cultivated after confluency and differentiated to enterocytes-like cells on Transwell^®^ support (Sigma Aldrich S.r.l., Milan, Italy) after 17–21 days, in line with many other articles (e.g., [24]). During the differentiation period, FBS-free media were added to the apical compartment, while complete medium was added to the basolateral compartment, every other day.

Human monocytic leukemia cells (THP-1; ATCC, Teddington, UK) were cultured in suspension with RPMI medium containing 100 mg streptomycin, 100 units penicillin, 4 mM L-glutamine, and 10% FBS. After 48 h or 72 h, cells were centrifuged, counted, and placed into new 75 cm^2^ flask or experimental supports. In the latter case, PMA 25 μM was added to complete medium before treatments to obtain adherent macrophages after 48 h.

For all treatments, FBS-free medium was used.

### 2.3. Cell Viability

Cell morphology was observed at the end of each experiment by light microscope inspection. In addition, cell viability was measured by 3,4,5-dimethylthiazol-2-yl-2-5-diphenylte-trazolium bromide (MTT) assay, as previously described [22]. In brief, MTT solution (5 mg/mL in PBS) was diluted (25X) and added to adherent cells at the end of each treatment. Then, formazan salts were solubilized by isopropanol:DMSO (90:10 *v*/*v*) solution after 15–30 min of metabolization. Finally, the absorbance was read at 550 nm (VICTOR X3; PerkinElmer, Milano, Italy).

### 2.4. Evaluation of Chemokine Release

CaCo-2 cells seeded in 24-well plates (3 × 10^4^/well) were stimulated by the combination of IL-1β (10 ng/mL), IFN-γ (10 ng/mL), and in-vitro-digested gliadin (Ga) (1 mg/mL) for 24 h, as previously reported [22,23]. The release of IL-8, CXCL-10, and MCP-1 was measured by ELISA assay in cell media after simultaneous treatment with glucoraphanin or sulforaphane. The same method was also applied for the quantification of MCP-1 and IL-1β in sample media collected from Transwell^®^ plates.

The ELISA assay was performed as previously described [22], following manufacturer instructions (PeproTech, London, UK). In brief, EIA/RIA plates (Merck Life Science, Milano, Italy) were coated with the capture antibodies and kept at room temperature overnight. Then, 100 μL of sample was added and compared with a standard calibration curve (0–1500 pg/mL). The number of chemokines was measured after the addition of biotinylated primary antibody and avidin-horseradish peroxidase enzyme, by the acquisition of the absorbance value resulting from the colorimetric reaction with 2,20-azino-bis(3-ethylbenzothiazoline-6-sulfonic acid) (ABTS) substrate (Merck Life Science, Italy). The absorbance was read using a spectrophotometer (VICTOR X3; PerkinElmer, Milano, Italy) at 405 nm. Data (mean ± SEM of at least three experiments) were expressed as a percentage relative to the stimulated control, which was arbitrarily assigned the value of 100%.

Apigenin (20 μM) or sodium butyrate (2 mM) were chosen as reference anti-inflammatory compounds, according to previously published papers related to intestinal inflammation [25,26].

### 2.5. Measurement of Epithelial Integrity

Epithelial integrity was evaluated by measuring the trans-epithelial electrical resistance (TEER) opposed by CaCo-2 cells monolayer (enterocyte-like), upon differentiation on 12-well Transwell^®^ support (3 × 10^5^/well). Three concordant TEER values (Ω) were collected by EVOM3 device (WPI, Sarasota, FL, USA) at the beginning (t0) of each experiment, with cut-off value above 400 Ω.

To observe the modulation of epithelial integrity, we applied two different types of inflammatory damage to the basolateral compartment, which simulated the intestinal lamina propria: (a) the pro-inflammatory combination including digested gliadin (IL-1β, IFN-γ, Ga); (b) previously cultivated THP-1 macrophages in 12-well plate (2 × 10^5^/well), stimulated with LPS (100 ng/mL) and IFN-γ (10 ng/mL). The molecules under study were simultaneously added to the apical side for 24 h, thus mimicking the exposure through the diet. Sodium butyrate (2 mM) was used as reference trophic factor for the gut barrier [26].

Finally, the variation in TEER values (ΔΩ) was calculated at the end of each experiment (t24h) as follows: ΔΩ = Ωt24h − Ωt0. Results were normalized on the stimulated condition, which was arbitrarily assigned to the value of 0. Thus, data were expressed as the mean of normalized ΔΩ ± SEM.

### 2.6. Luciferase Assay and Transcription Factors

CaCo-2 cells were seeded in 24-well plates (3 × 10^4^/well) for 48 h, then transfected with plasmids containing elements responsive to κB (100 ng per well) or Nrf-2 (200 ng per well). The activity of both transcription factors was evaluated after 6 h of treatment with molecules under study and pro-inflammatory (IL-1β, IFN-γ, Ga) or pro-oxidant (H_2_O_2_ 1 mM) stimuli. NF-κB plasmid was a gift from Dr. N. Marx (Department of Internal Medicine-Cardiology, University of Ulm; Ulm, Germany), while Nrf-2 was shared by O’Connell et al. [27] through the Addgene non-profit repository (#90398, Addgene, LGC Standards, Teddington, UK). As previously reported [22], CaCo-2 cells were transiently transfected by Lipofectamine^®^ 3000 Reagent, following manufacturer instructions (Life Technologies Italia, Segrate, Italy). Britelite^TM^ Plus reagent, containing luciferin, was used to assess the amount of luciferase produced in the cells at the end of the treatment. A plate reader (VICTOR X3, Perkin Elmer, Milan, Italy) was used to measure luminescence deriving by the reaction between luciferin and luciferase. Resveratrol and apigenin (20 μM) were used as reference antioxidant and anti-inflammatory natural compounds, respectively.

### 2.7. Expression and Activity of Catalase

To evaluate catalase (CAT) expression and enzymatic activity, CaCo-2 cells were cultivated in 24-well plates (3 × 10^4^/well) for 48 h, then treated with glucoraphanin or sulforaphane (25 μM) in addition to the pro-oxidant stimulus H_2_O_2_ (1 mM) for 6 h. The mRNA was extracted with NucleoSpin^®^ RNA plus kit (Macherey-Nagel GmbH & Co, Düren, Germany) and quantified by spectrophotometric analysis at 260 nm (NanoDrop ND-100 spectrophotometer, Euroclone, Italy). The amplification of the gene coding for human catalase (hCAT) was assessed by rt-PCR using forward (CAGATAGCCTTCGACCCAAG) and reverse primers (TTTGCCTATCCTGACACTCACCGC), purchased from Eurofins (Eurofins Scientific, Milan, Italy).

Real-time PCR reaction was conducted on 10 ng mRNA/well in the Real-Time System Bio-Rad CFX384 (Bio-Rad, CA, USA) using iTaqTM Universal SYBR Green One-Step Kit (Bio-Rad). The thermal cycling protocol required a preliminary step for reverse transcription at 50 °C for 10 min, followed by polymerase activation step (95 °C, 5 min) and 40 cycles with 95 °C denaturation step for 10 s. and annealing/extension at 60 °C for 30 s. All samples were tested in triplicate, and the relative expression of hCAT genes was calculated by normalizing the threshold cycle (Ct) with the Ct of 36B4 gene expression, to correct for variation in RNA loading.

Catalase activity was measured by colorimetric assay, following manufacturer’s instructions (Cayman Chemical, MI, USA), as previously described [28]. In brief, cell lysate samples were obtained through lysis buffer (K_2_PO_4_ 50 mM, pH 7.0, EDTA 1 mM, Triton-X 0.1%); then, protein content was measured by BCA assay, to normalize the amount of sample for the enzymatic test. The CAT activity of each sample (10 μg) was measured by referring to the capacity to oxidize methanol into formaldehyde (CAT peroxidatic activity), determined by the colorimetric reaction of the latter with 4-amin-3-hydrazino-5-mercapto-1,2,3-triazole (Purpald). Purpald leads to an adduct with absorbance at 535 nm (EnVision; PerkinElmer, Milano, Italy). The quantitative data were obtained by comparison with a calibration curve of formaldehyde (0–75 μg/mL). The absorbance values were normalized on total protein content and expressed as CAT activity on μg of sample proteins (mean ± SEM).

### 2.8. Phosphorylation of Histone γ-H2AX

CaCo-2 cells were cultivated in 24-well plates (10^4^/well) for 24 h, in which microscope slides were previously placed. Then, cells were treated with glucoraphanin or sulforaphane (25 μM) in addition to the pro-oxidant stimulus H_2_O_2_ (1 mM) for 1h. The degree of DNA damage at the nuclear level was investigated by immunostaining the phosphorylated form of histone γ-H2AX (ser 139), as a well-known marker of double-strand break and DNA repair initiation.

The immunofluorescence technique was performed by using anti-γ-H2AX (ser 139) rabbit antibody (#9718T, Cell signaling, MA, USA), combined with AlexaFluor^®^ 594-conjugated anti-rabbit antibody (# 8889S Cell signaling, MA, USA), according to manufacturer instructions. In brief, samples were fixed using PBS 1X/PFA 4% solution for 15 min; then, samples were washed with PBS 1X, and blocking–permeabilizing solution was added (BSA 5%, Triton-X 0.3%, in PBS 1×) for 1 h. Primary antibody against γ-H2AX was added after dilution (1:1000, 1 μg/mL), and left at 4 °C until the day after, when anti-rabbit conjugate antibody was added (dilution 1:2000) for 1 h. Finally, cells were washed three times and mounted on glass slides with ProLong Gold Antifade DAPI (Cell Signaling, MA, USA). Images were acquired by confocal microscopy (LSM 900, Zeiss, Oberkochen, Germany).

### 2.9. LC-MS/MS Analysis on Broccoli Sprouts Sample

Broccoli sprouts collected from the market (Vivo S.r.l. Società Agricola, Naturalsalus S.r.l) were milled by mortar and kept at room temperature for 1 h, to allow the conversion of glucoraphanin into sulforaphane. Then, homogenized material was extracted with hydroalcoholic solution (50:50 ethanol:water), as described in [22] with minor modifications. In brief, 10 g of plant material was extracted twice for 4 h and 16 h with 100 mL of solvent. The extract obtained was concentrated by Rotavapor (Heidolph Instruments GmbH & CO, Schwabach, Germany), freeze-dried (Edwards, 5Pascal, Trezzano, Italy), and kept at −20 °C. The yield of extraction was 5.14% of the weight of dry extract on fresh plant material.

LC-MS/MS analysis was performed on broccoli sprouts extract before and after the simulation of the gastro-intestinal digestion, following previously described protocols [22]. In brief, HPLC was performed through an Exion LC^TM^ AC System (AB Sciex, Foster City, CA, USA) composed of a vacuum degasser, a double plunger pump, a cooled autosampler, and a temperature-controlled column oven. The MS/MS analysis was carried out with a Triple Quad^TM^ 3500 system (AB Sciex, Foster City, CA, USA). The analytes were separated on a Synergi 4 um Hydro-RP 80 A LC Colum 150 × 4.6 mm (Phenomenex, Torrance, CA, USA) with a mobile phase composed of 0.1% formic acid in water (A) and methanol 50:50 (B) at a flow rate of 0.600 mL/min. The chromatographic gradient was set as reported in Table 1.

The injection volume was 10 μL for each sample. Mass spectrometric detection was done in negative ionization (ESI) mode for glucoraphanin and in positive ionization (ESI) mode for sulforaphane. The optimized compound-dependent MS/MS parameters (declustering potential, entrance potential, collision energy, and collision cell exit potential) were obtained, in multiple-reaction-monitoring (MRM) mode, by a separate infusion of the analytes. The analytes were quantified by using the following mass transitions: 178/114 (sulforaphane), 436/97 (glucoraphanin), using pure standard commercially available.

The LC–MS/MS system was controlled by AB Sciex Analyst^®^ (version 1.7) software.

### 2.10. Measurement of the Antioxidant Capacity through DPPH and FRAP Assays

The antioxidant capacity of the extracts was evaluated spectrophotometrically by using the 1,1-diphenyl-2-picryl-hydrazyl (DPPH) assay and the Ferric Reducing Antioxidant Power (FRAP) assay, as previously described [29].

For the assay, 150 μL of 0.005% DPPH in methanol was mixed with 50 μL of each prepared sample. The absorbance was then measured at 517 nm after 30 minutes using an Enspire^®^ Multimode plate reader (PerkinElmer, Waltham, MA, USA). The scavenger concentration was determined using a calibration curve of gallic acid, with concentrations ranging from 1.0 to 5.0 μg/mL (equivalent to 6 to 30 μM) and expressed as gallic acid equivalents (GAE) in μM.

In the FRAP test, the oxidized and colorless form of iron, Fe^3+^, is converted by antioxidant compounds into its reduced form, Fe^2+^. In the presence of 2,4,6-tripyridyl-s-triazine (TPTZ), a blue TPTZ-Fe^2+^ complex is formed, which has a characteristic absorption peak at 593 nm. The antioxidant capacity was determined using a standard curve of FeSO_4_ (ferrous sulfate heptahydrate), with concentrations ranging from 0.11 to 0.75 mM. The FRAP reagent was prepared by combining 300 mM acetate buffer, 10 mM TPTZ solution (2,4,6-tripyridyl-s-triazine), and 20 mM FeCl_3_ (7H_2_O) solution (ferric chloride hexahydrate) in a ratio of 10:1:1 (*v*/*v*/*v*). Portions of 5 μL of standard solution, sample, or blank were mixed with 15 μL of HPLC-grade water and 150 μL of FRAP reagent, then incubated at 37 °C for 30 minutes in the dark. The absorbance was then measured at 593 nm using an Enspire^®^ Multimode plate reader (PerkinElmer, Waltham, MA, USA). The results were expressed as concentration of Fe^2+^ (mM) reduced by tested substances, equivalent to FeSO_4_ (7H_2_O) standard. 

### 2.11. Statistical Analysis

All biological results are expressed as the mean ± SEM (standard error), while analytical data were expressed as mean ± SD (standard deviation) of at least three independent experiments. Data were analyzed by unpaired ANOVA test followed by Bonferroni post hoc analysis. Statistical evaluation and IC_50_ calculation were performed using GraphPad Prism 8.0 software (GraphPad Software Inc., San Diego, CA, USA). Values at *p* < 0.05 were considered statistically significant.

## 3. Results

### 3.1. The Effect of Glucoraphanin and Sulforaphane on Inflammatory and Oxidative Markers in CaCo-2 Cells

In our previous works [22,23], we observed that the addition of digested gliadin (1 mg/mL) to IL-1β and IFN-γ (10 ng/mL) enhanced the release of the chemokines IL-8, CXCL-10, and MCP-1 by CaCo-2 cells. Thus, the same experimental protocol was applied at the beginning of this study, to compare the biological activity of glucoraphanin and sulforaphane.

**Figure 1:**
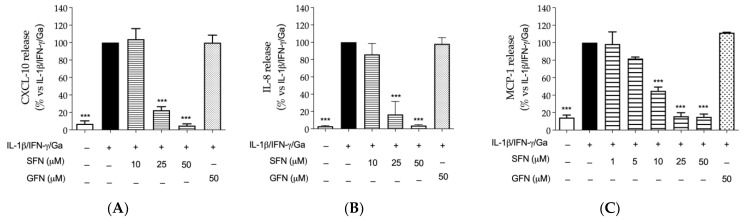
Effect of sulforaphane and glucoraphanin on the release of chemokines by CaCo-2 cells. The release of CXCL-10 (**A**), IL-8 (**B**), and MCP-1 (**C**) was measured by ELISA assay. Cells were treated with sulforaphane (SFN, horizontal lines) or glucoraphanin (GFN, dots), in addition to the pro-inflammatory combination of IL-1β, IFN-γ (10 ng/mL), and digested gliadin (Ga, 1 mg/mL) (black bar), for 24 h. Apigenin 20 μM was used as reference inhibitor of CXCL-10 (−30%), IL-8 (−16%), and MCP-1 (−30%). Data from independent experiments (*n* = 3) were reported as mean of release (% ± SEM) vs. stimulus, to which was arbitrarily attributed the value of 100%. *** *p* < 0.001 vs. stimulus.

**Figure A1:**
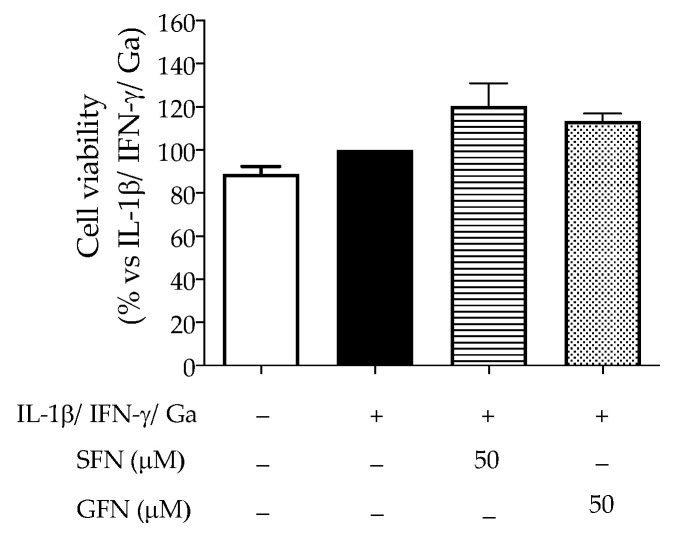
Cell viability measured by MTT assay. CaCo-2 cells were treated with sulforaphane (SFN, horizontal lines) or glucoraphanin (GFN, dots), in addition to the pro-inflammatory combination of IL-1β, IFN-γ (10 ng/mL), and digested gliadin (Ga) (black bar), for 24 h. Data from independent experiments (*n* = 3) were reported as mean absorbance (% ± SEM) vs. stimulus, to which was arbitrarily attributed the value of 100%.

Sulforaphane inhibited the release of all selected chemokines at concentrations below 50 μM (Figure 1A–C). On the contrary, glucoraphanin showed no inhibitory effect at 50 μM. The inhibitory effect of sulforaphane showed a concentration-dependent fashion, being more pronounced against MCP-1 release (IC_50_ of 7.81 μM). Cell viability was not significantly altered by the molecules under study (Figure A1).

The inflammatory process has been associated with oxidative stress and DNA damage in intestinal samples from celiac patients [15,19,20,21]. Monguzzi and colleagues suggested that digested gliadin itself (500 μg/mL) might induce a small increase in ROS production and phosphorylation of histone γ-H2AX (ser 139) in CaCo-2 cells, the latter being a well-recognized marker of genotoxicity [19].

In our experimental model, we could only attribute irrelevant effects to digested gliadin (1 mg/mL) through the evaluation of oxidative markers (γ-H2AX, ROS) during 24 h of treatment. For this reason, we carried out the following experiments by using H_2_O_2_ to reproduce a pro-oxidant and genotoxic context.

Thus, the phosphorylation of γ-H2AX was selected as an early marker of DNA damage (1 h), while the activity of Nrf-2 and catalase was selected as later read-outs of oxidative stress (6 h). In parallel, the direct scavenging capacity of the compounds was addressed by cell-free systems, such as DPPH and FRAP assays.

**Figure 2:**
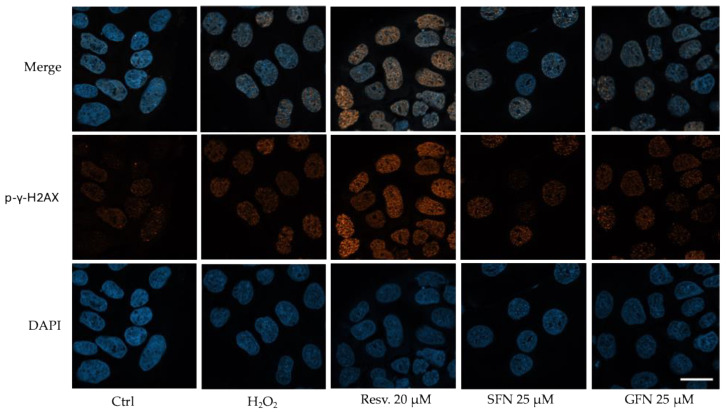
Effect of sulforaphane and glucoraphanin on DNA damage caused by H_2_O_2_ in CaCo-2 cells. DNA double-strand break was revealed by immunofluorescence staining of phospho-γ-H2AX (ser 139). Cells were treated with sulforaphane (SFN 25 μM) or glucoraphanin (GFN 25 μM) for 1 h, in addition to the pro-oxidant stimulus H_2_O_2_ (1 mM). Resveratrol 20 μM (Resv.) was used as reference antioxidant compound. Representative images from independent experiments (*n* = 3) were reported (60× objective magnification, scale bar equivalent to 50 μm).

Neither sulforaphane nor glucoraphanin (25 μM) altered the phosphorylation of γ-H2AX induced by H_2_O_2_, which caused a clear increase in the number of nuclear foci, as expected (Figure 2). Resveratrol 20 μM, selected as a reference antioxidant compound for all the experiments concerning oxidative stress, caused an additional increase in γ-H2AX phosphorylation; this observation probably reflected the enhancement of DNA repair systems and apoptotic signals, which is considered positive against cancer progression [30]. A further indication was obtained by using a direct scavenger like ascorbate (100 μM), which showed results identical to resveratrol in our experimental settings.

In line with these data, sulfur compounds exhibited no scavenging power in the redox assays DPPH and FRAP at the concentration of 200 μM, in comparison to resveratrol 100 μM (Table 2).

Data were reported as mean concentration (μM) of GAE required to scavenge DPPH radicals, or FeSO_4_ equivalents of reduced FeCl_3_ ± S.D, respectively. GAE, gallic acid equivalents; SFN, sulforaphane; GFN, glucoraphanin; Resv., resveratrol; n.d., not detectable.

During oxidative damage, the phosphorylation of γ-H2AX is required to trigger not only pathways responsible for DNA repair but also the antioxidant defense in which Nrf-2 is involved [31]. Thus, the transcriptional activity of Nrf-2 was measured in correlation with catalase levels, to depict a more complete picture of the potential antioxidant properties of sulfur compounds.

**Figure 3:**
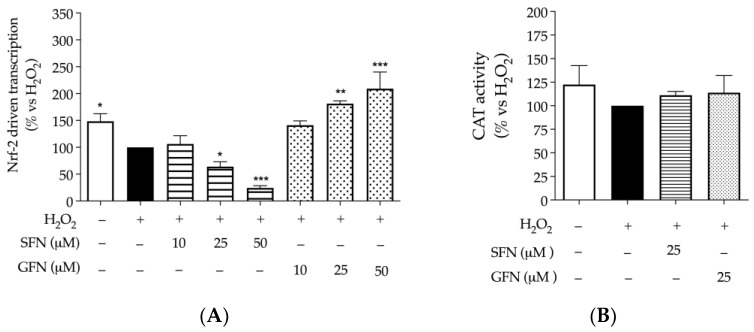
Effect of sulforaphane and glucoraphanin on Nrf-2 driven transcription and catalase activity in CaCo-2 cells. The activity of Nrf-2 was measured after plasmid transfection by luciferase assay (**A**), while catalase activity was measured by enzymatic assay (**B**). Cells were treated with sulforaphane (SFN, horizontal lines) or glucoraphanin (GFN, dots) for 6 h in addition to the pro-oxidant stimulus H_2_O_2_ 1 mM. Resveratrol 20 μM was used as reference inducer of Nrf-2 (+220%). Data from independent experiments (*n* = 3) were reported as mean activity (% ± SEM) vs. stimulus (black bar), to which was arbitrarily attributed the value of 100%. * *p* < 0.05, ** *p* < 0.01, *** *p* < 0.001 vs. stimulus.

H_2_O_2_ (1 mM) caused a small decrease in both Nrf-2 and catalase activity, although significant only for the first. Similar data were obtained by other authors in HepG2 cells challenged with H_2_O_2_ 1 mM [32]. Surprisingly, sulforaphane further impaired the activity of Nrf-2 in a concentration-dependent fashion, while glucoraphanin showed the opposite effect (Figure 3A). Again, resveratrol, used as a reference antioxidant at a comparable concentration (20 μM), increased the activity of Nrf-2 by more than twofold with respect to H_2_O_2_.

**Figure A2:**
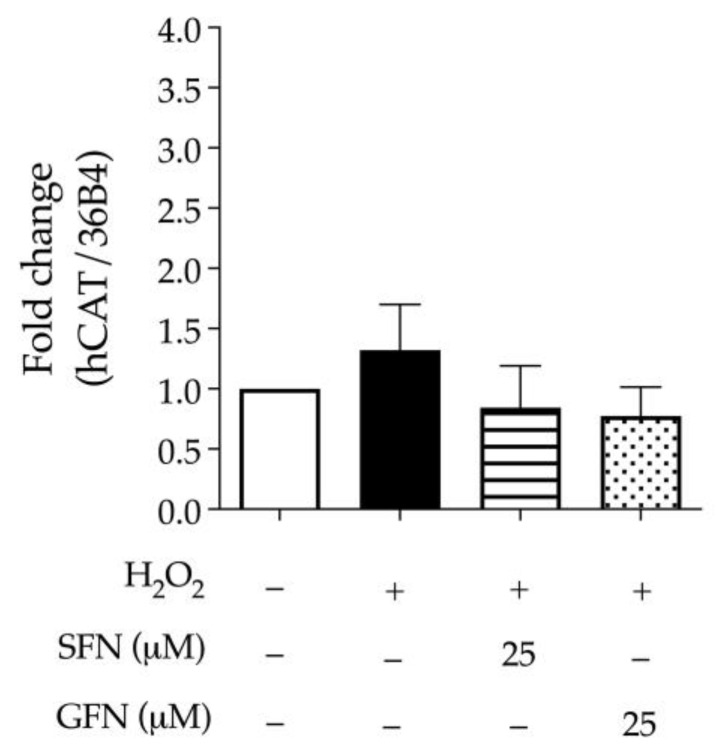
Gene expression of catalase gene (hCAT) measured by rt-PCR in CaCo-2 cells. Cells were treated with sulforaphane (SFN, horizontal lines) or glucoraphanin (GFN, dots), in addition to the pro-oxidant stimulus H_2_O_2_ (1 mM) (black bar), for 6 h. Data from independent experiments (*n* = 3) were reported as fold change (% ± SEM) vs. untreated control (white bar), to which was arbitrarily attributed the value of 1.

This observation was not reflected in a relevant modulation of catalase, since neither enzymatic activity (Figure 3B) nor gene expression (Figure A2) were significantly altered by H_2_O_2_ or sulfur compounds. Of note, the same was observed after treating cells with resveratrol.

For a better comprehension of our findings, the interference with cell viability was excluded by the MTT test at 24 h, thus supporting the concept that H_2_O_2_ and pure molecules could modulate the activity of Nrf-2 through mechanisms independent of cytotoxic or antiproliferative effects.

The Nrf-2 pathway is widely considered one of the main targets of sulforaphane, even in experimental works dedicated to colitis [10,11]. It is known that Nrf-2-dependent signals might also explain the anti-inflammatory activity of sulforaphane through NF-κB impairment, although the precise mechanisms of action are still unclear. Nevertheless, the role of glucoraphanin is widely considered negligible.

The observation that both molecules modulated the activity of Nrf-2 in our model prompted further investigations concerning the potential consequence on the NF-κB pathway. With this in mind, the activity of NF-κB was measured by luciferase assay under both inflammatory and oxidative conditions.

**Figure 4:**
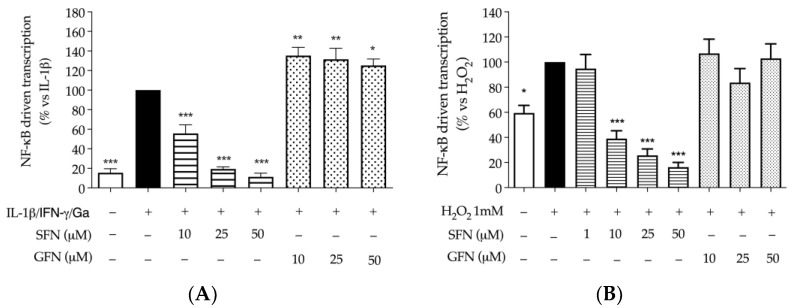
Effect of sulforaphane and glucoraphanin on NF-κB driven transcription in CaCo-2 cells. The activity of NF-κB was measured after plasmid transfection by luciferase assay. Cells were treated with sulforaphane (SFN, horizontal lines) or glucoraphanin (GFN, dots) for 6 h in addition to the pro-inflammatory combination of IL-1β, IFN-γ (10 ng/mL) and digested gliadin (Ga, 1 mg/mL) (**A**), or pro-oxidant stimulus H_2_O_2_ 1 mM (**B**). Apigenin 20 μM and resveratrol 20 μM were used as reference inhibitors (−97% and −90%, respectively). Data from independent experiments (*n* = 3) were reported as mean luminescence (% ± SEM) vs. stimulus (black bar), to which was arbitrarily attributed the value of 100%. * *p* < 0.05, ** *p* < 0.01, *** *p* < 0.001 vs. stimulus.

As supposed, sulforaphane showed inhibitory activity on the NF-κB driven transcription, with a concentration-dependent effect. In fact, the NF-κB driven transcription induced by the pro-inflammatory combination was impaired with an IC_50_ of 9.75 μM; glucoraphanin showed the opposite effect with respect to sulforaphane, since it slightly increased the activity of NF-κB at all the concentrations tested (Figure 4A). These data paralleled the results concerning the release of chemokines, described in Figure 1.

Sulforaphane also exhibited an inhibitory effect when NF-κB was stimulated by H_2_O_2_ (Figure 4B); however, the transcriptional activity was reduced under the basal level of unstimulated control, showing a parallelism with previous data obtained on Nrf-2. On the contrary, GFN was unable to modulate the activity of NF-κB in this context.

For a better comparison of the inhibitory concentrations measured for different inflammatory markers, IC_50_ was calculated, when possible, and is summarized in Table 3.

Ga, in-vitro-digested gliadin (1 mg/mL).

At this point of our work, we wondered whether the biological activity might implicate a protective effect on intestinal barrier integrity, which is impaired in celiac disease [13,14]. For this purpose, sulforaphane and glucoraphanin were investigated in enterocyte-like differentiated CaCo-2 cells, cultivated on Transwell^®^ support.

Aiming at dissecting a direct action on the epithelial barrier with respect to an indirect effect involving immune cells, experiments were conducted with two different settings: (a) stimulation of CaCo-2 with the pro-inflammatory combination of IL-1β, IFN-γ and digested gliadin; (b) co-culture of CaCo-2 with THP-1 macrophages, stimulated by LPS and IFN-γ. Experiments dedicated to the experimental settings excluded that gliadin alone, or the combination of LPS with IFN-γ in the absence of macrophages, might impair the epithelial integrity.

**Figure 5:**
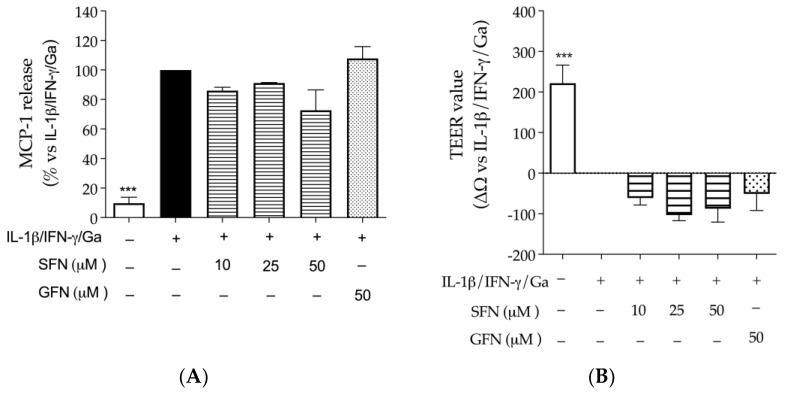
Effect of sulforaphane and glucoraphanin on CaCo-2 epithelial barrier (Transwell^®^ model). Cells were treated with sulforaphane (SFN, horizontal lines) or glucoraphanin (GFN, dots) for 24 h, in addition to the pro-inflammatory combination of IL-1β, IFN-γ (10 ng/mL), and digested gliadin (Ga, 1 mg/mL): (**A**) The release of MCP-1 was measured by ELISA assay. Data from independent experiments (*n* = 3) were reported as mean release (%) ± SEM vs. stimulus (black bar), to which was arbitrarily attributed the value of 100%. (**B**) Epithelial integrity was measured as normalized TEER variation (ΔΩ = Ωt24h − Ωt0). Data from independent experiments (*n* = 4) were reported as normalized ΔΩ ± SEM vs. stimulus, to which was arbitrarily attributed the value of 0. Sodium butyrate 2 mM was used as reference inhibitor of MCP-1 release (−40%) and trophic factor for the epithelial barrier (+40 Ω). *** *p* < 0.001 vs. stimulus.

Regarding the first experimental setting, sulforaphane (10, 25, 50 μM) and glucoraphanin (50 μM) were neither able to reduce the release of MCP-1 (Figure 5A), selected as an inflammatory outcome, nor restore the epithelial integrity (TEER values), compromised by the pro-inflammatory combination of IL-1β, IFN-γ, and digested gliadin (Figure 5B).

**Figure 6:**
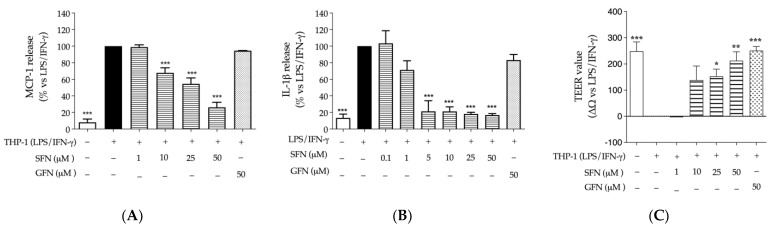
Effect of sulforaphane and glucoraphanin on the epithelial barrier in the co-culture of CaCo-2 and THP-1 macrophages (Transwell^®^ model). Cells were treated with sulforaphane (SFN, horizontal lines) or glucoraphanin (GFN, dots) for 24 h, in addition to the pro-inflammatory combination of LPS (100 ng/mL) and IFN-γ (10 ng/mL): (**A**,**B**) The release of MCP-1 and IL-1β was measured by ELISA assay on media collected from co-culture or THP-1, respectively. Data from independent experiments (*n* = 3) were reported as mean release (%) ± SEM vs. stimulus (black bar), to which was arbitrarily attributed the value of 100%. (**C**) Epithelial integrity was measured as normalized TEER variation (ΔΩ = Ωt24h − Ωt0). Data from independent experiments (*n* = 4) were reported as normalized ΔΩ ± SEM vs. stimulus, to which was arbitrarily attributed the value of 0. Sodium butyrate 2 mM was used as reference inhibitor of MCP-1 release (−17%) and trophic factor for the epithelial barrier (+220 Ω). Apigenin 20 μM was used as reference inhibitor of IL-1β (−72%). * *p* < 0.05, ** *p* < 0.01, *** *p* < 0.001 vs. stimulus.

Regarding the second experimental setting, sulforaphane was able to impair the release of MCP-1 and IL-1β, with an IC_50_ of 20.60 μM and 1.50 μM, respectively (Figure 6A,B). It also restored the epithelial integrity at concentrations ranging from 10 to 50 μM, with an effect comparable to the reference compound sodium butyrate, used at higher concentrations (2 mM) (Figure 6C).

Of note, glucoraphanin, selected at the concentration of 50 μM, showed similar protective effects on the epithelial barrier, without showing anti-inflammatory properties.

### 3.2. Quantification of Sulforaphane before and after In Vitro Gastrointestinal Digestion of the Extract

Since sulforaphane showed the most interesting biological activity, we decided to verify its possible stability at the small intestine level, with an estimation from a complex extract mixture derived from a dietary source of sulforaphane, *Brassica oleracea*. Broccoli sprouts collected from the market were processed and extracted with the aim of obtaining the complete conversion of glucoraphanin into sulforaphane. Then, the extract underwent a simulated gastrointestinal digestion, as previously reported [22], and then HPLC-MS was used for quantitative analysis.

HPLC-MS showed that sulforaphane was present in the extract, while glucoraphanin was absent. The amount of sulforaphane was slightly increased after digestion, moving from 385 μg/g plant material before in vitro gastrointestinal digestion to 503 μg/g plant material after the digestion process. These data suggest that sulforaphane eventually present in the vegetable matrix might remain stable through the gut lumen, in line with the literature published in this regard [11].

## 4. Discussion

Glucoraphanin, a glucosinolate present in cruciferous vegetables, is considered an inert precursor of sulforaphane, which exerts a plethora of biological activities in animal models and human studies, including antioxidant and anti-inflammatory [2,3,4,5].

Even though glucoraphanin has been reported to pass through the gut lumen in stable form until reaching the colon microbiota [2], its biological activity, and that of sulforaphane, are poorly investigated with reference to the first tract of the digestive system. Accordingly, the aim of this study was to compare the biological activity of sulforaphane with its precursor glucoraphanin in a cellular model of gliadin-induced inflammation, to suggest a possible dietary role in the framework of celiac disease. For this purpose, selected concentrations were achievable after vegetable consumption, namely, 1 to 50 μM [2].

Sulforaphane inhibited the release of all selected chemokines IL-8, CXCL-10, and MCP-1, which have crucial role in leukocytes recruitment; of note, a stronger effect on the monocyte’s attractor MCP-1 (IC_50_ of 7.81 μM) was observed, even in comparison with the reference inhibitor apigenin (Figure 1). On the contrary, glucoraphanin was unable to reduce the release of chemokines. The inhibitory mechanism of sulforaphane was attributed to the NF-κB pathway, which was impaired at comparable concentrations (IC_50_ of 9.75 μM) (Figure 4A).

Regarding markers of oxidative stress, molecules showed no effect on γ-H2AX phosphorylation at the concentration tested of 25 μM (Figure 2), contrary to resveratrol, whose effect was interpreted as anti-tumoral according to previous works [30]. The result could be explained, at least in part, by the lack of direct scavenging capacity observed in DPPH and FRAP assays, again, in contrast with the properties of resveratrol (Table 2). However, similar conclusions regarding the antioxidant activity of sulforaphane were previously reported by other authors, who suggested that biological rather than scavenging mechanisms are involved [33]. In fact, later events typical of oxidative stress, such as Nrf-2 and NF-κB activation, were modulated by compounds, including glucoraphanin, thus supporting the concept that molecules could act through biological pathways after cellular uptake. More precisely, the two molecules exhibited divergent effects on the two transcription factors, which were generally inhibited by sulforaphane, while glucoraphanin showed negligible effect on NF-κB and induced the activity of Nrf-2 without affecting catalase levels (Figure 3A and Figure 4A,B).

It is evident that the antioxidant effect exerted by pure glucosinolate and its metabolite is more complex to explain with respect to the anti-inflammatory one. A possible limitation might regard the use of tumoral cells, which is the case of CaCo-2, since DNA damage and Nrf-2 modulation are mechanisms shared by antiproliferative compounds, such as resveratrol and sulforaphane [34,35]. Moreover, sulforaphane is known to induce Nrf-2 and inhibit NF-κB through complex pathways, including, among others, enhanced H_2_S production and HDAC inhibition [36,37]. On the contrary, the mechanism of action of glucoraphanin was less characterized, thus representing an element of novelty.

In consideration of the above, it is our opinion that the experimental setting adopted to study oxidative stress, based on the use of H_2_O_2_ (1 mM) in comparison to resveratrol (20 μM), allowed several hypotheses about compounds under study to be inferred. Within the limits of our settings, resveratrol increased the phosphorylation of γ-H2AX, increased the activity of Nrf-2, and decreased the activity of NF-κB, thus reflecting the framework of DNA repair and antioxidant defense [31]. The bioactivity of glucoraphanin and sulforaphane was neither comparable to resveratrol nor superimposable. In fact, they showed divergent effects on inflammatory and oxidative markers.

In differentiated CaCo-2 cells, cultivated on Transwell^®^ support, the anti-inflammatory activity of sulforaphane was still observable during co-culture with activated macrophages (THP-1). In fact, the release of MCP-1 and IL-1β was reduced at low μM concentrations (Figure 6). Accordingly, glucoraphanin was not active in this inflammatory context. However, both molecules recovered the trans-epithelial electrical resistance (TEER), thus suggesting a role for glucoraphanin at least in the protection of epithelial barrier integrity. Consequently, NF-κB and HDAC are recognized targets of sulforaphane, but they are also influenced by trophic factors for intestinal mucosa, such as butyrate, used as a reference compound in our experiments [26]. To the best of our knowledge, the structure–activity relationship of glucoraphanin with respect to HDAC isoforms is still unexplored.

Conversely, the effect on barrier function was absent when the Transwell^®^ model was elicited by the pro-inflammatory cocktail containing gliadin, thus remarking that different mechanisms underneath epithelial damage occurred in our experimental conditions. In the first case, mediators from macrophages, which plausibly include protease, oxidative insults, and inflammatory signals, were necessary to induce epithelial damage. In fact, LPS and IFN-γ, namely, the triggers of macrophage activation, were unable to cause TEER alterations when used alone. In the second case, direct damage was caused by cytokines (IL-1β, IFN-γ) and gliadin to the epithelial barrier. Thus, it is plausible that the activity of sulforaphane and glucoraphanin on barrier protection might result from an anti-inflammatory effect on macrophages; in line with our hypothesis, this mechanism has already been reported for sulforaphane by other authors [38].

Consequently, further experiments should be devoted to a deep comprehension of the mechanisms retained by the molecular structure of glucoraphanin with respect to sulforaphane.

## 5. Conclusions

To the best of our knowledge, this is one of the first articles in which sulforaphane and glucoraphanin were considered as dietary compounds with potential interest for healthy diets dedicated to celiac people [39]. The investigation of the biological activity of sulforaphane in intestinal epithelial cells, with a focus on gliadin-induced inflammation, is an element of novelty; moreover, few authors have considered the comparison with its precursors before [37], which, in our opinion, should not be considered exclusively as a pro-active compound.

The anti-inflammatory and antioxidant activity of sulforaphane was demonstrated at concentrations close to those available after oral administration in vivo [2,38]. Moreover, our work gained more insight into the biological effect of glucoraphanin on the first tract of the gut lumen, with repercussions on aspects related to the quality of cruciferous plant-based products present in food and pharmaceuticals.

## Figures and Tables

**Table 1:** Chromatographic gradient.

Time (min)	Phase A (%)	Phase B (%)
0	90	10
1	90	10
6	0	100
8	0	100
8.10	90	10
10	90	10

**Table 2:** Scavenging capacity of sulforaphane and glucoraphanin, measured by DPPH and FRAP tests.

	DPPH (μM GAE ± S.D.)	FRAP (μM FeSO_4_ ± S.D.)
SFN (200 μM)	n.d.	n.d.
GFN (200 μM)	n.d.	n.d.
Resv. (100 μM)	26.63 ± 2.37	333.98 ± 9.67

**Table 3:** Summary of the IC_50_ of sulforaphane on inflammatory markers measured in CaCo-2 cells stimulated with a pro-inflammatory cocktail including digested gliadin (IL-1β, IFN-γ, Ga).

Inflammatory Markers	IC_50_ (μM) of Sulforaphane
CXCL-10	23.55
IL-8	15.74
MCP-1	7.81
NF-κB	9.75

## Data Availability

Data are contained within the article.
